# Osmotic Dehydration of Apples in a Saccharose Solution Containing Fragrant Agrimony or Rosehip Extract

**DOI:** 10.3390/molecules30244708

**Published:** 2025-12-09

**Authors:** Elżbieta Karlińska, Joanna Milala, Monika Kosmala, Robert Klewicki

**Affiliations:** Institute of Food Technology and Analysis, Lodz University of Technology, Stefanowskiego Street 2/22, 90-537 Lodz, Poland; joanna.milala@p.lodz.pl (J.M.); monika.kosmala@p.lodz.pl (M.K.); robert.klewicki@p.lodz.pl (R.K.)

**Keywords:** fragrant agrimony, *Agrimonia procera* Wallr., rosehip, *Rosa rugosa*, osmotic dehydration, polyphenols, ellagitannins, agrimoniin

## Abstract

In the present study, extracts from the fragrant agrimony (*Agrimonia procera* Wallr.) herb and the pseudo-fruits of rose (*Rosa rugosa*) were incorporated into a 50% sucrose solution used for the osmotic dehydration of Champion apples (*Malus domestica* Borkh.). This approach enabled the investigation of the migration of fragrant agrimony and rose polyphenols—both total polyphenols and their main representatives—during the dehydration process of apples, which are among the most popular fruits due to their health-promoting and nutritional properties. The total polyphenol content was determined using spectrophotometric methods, while the major individual compounds were quantified by UHPLC-DAD-MS. At a polyphenol content of 4 g/L in the solution, a more intensive water migration (water loss of about 3 g/g DM) from the fruit tissue was achieved for both extracts compared to the pure sucrose solution. However, no relationship between the polyphenol level in the hypertonic solution and the migration of sucrose into the apple tissue was observed. With regard to polyphenolic compounds, the level of polyphenols in apples dehydrated in the presence of extracts, compared to those dehydrated in pure sucrose solution, increased with the extract dose. The maximum value—approximately 825 mg/100 g DM of total polyphenols—was obtained at an extract concentration of 6 g/L, derived from both fragrant agrimony herb and rose pseudo-fruit. In the apples dehydrated using the extracts, the presence of phenolic compounds not found in fresh apples, characteristic of the applied extracts, such as ellagitannins, ellagic acid, flavonols including quercetin and kaempferol glycosides, as well as flavones, including derivatives of apigenin and luteolin, was observed. These findings indicate that the use of fragrant agrimony and rose extracts in osmotic dehydration may serve as an effective strategy for enhancing the polyphenolic profile and functional value of dehydrated apple products.

## 1. Introduction

A popular dehydrating agent used in osmotic dehydration is sucrose, applied in the form of a concentrated (50–60 °Bx) solution. Such a hypertonic solution draws water from the plant tissue. At the same time, the osmotic agent migrates into the material being dehydrated—sucrose modifies the nutritional and organoleptic properties of the product, acts as a preservative [1], Available evidence suggests that a range of bioactive compounds can be effectively introduced into raw materials, particularly fruits, through osmotic dehydration in a sucrose solution, provided that these compounds are incorporated into the osmotic medium alongside sucrose. For example, Nambiar et al. [2] transferred fructooligosaccharides [3,4] and vitamin C to passion fruit.

A group of compounds with valuable properties for the human body are polyphenols. They are indicated to have potentially beneficial effects in the context of health problems such as neurodegenerative diseases, inflammations, cancers, cardiovascular diseases, type 2 diabetes, and obesity [5]. From the perspective of food production, it is also important that polyphenols exhibit preserving properties [6].

Some data are available on the introduction of these substances into osmotically dehydrated plant material through the addition of juices or extracts from raw materials rich in polyphenols to a hypertonic solution or by using concentrated juices as a dehydrating agent. In this way, an extract from *Garcinia indica* was used to transfer anthocyanins to Indian gooseberry [7]. A product with improved physicochemical and sensory properties was obtained. An additional benefit was also the increased content of ascorbic acid. The above extract was also used in the research by Channannavar et al. [8] to transfer anthocyanins to pineapple. Meanwhile, Bellary et al. [9] performed an infusion into watermelon, obtaining a sensory-acceptable and stable product. Hernández-Carranza et al. [10] performed osmoconcentration in an extract from *Hibiscus sabdariffa* with the addition of sucrose, improving the antioxidant properties of apples and giving them a pleasant color. Cichowska et al. [11] used aronia juice concentrate to increase the polyphenol content in apples, which resulted in a significant presence of these compounds even after long-term storage of dehydrated fruit. Samborska et al. [12], in addition to aronia juice concentrate, also used bilberry extract, modifying characteristics such as color, taste, aroma, and softness of dehydrated apples. Bioactive compounds from blueberries were also introduced into apples (Castagnini et al. [13]) using the vacuum impregnation method in a juice. Polyphenols from grapes were transferred to mango (Medeiros et al. [14]) successfully testing the techniques of osmotic dehydration assisted atmospheric pressure impregnation and osmotic dehydration assisted vacuum impregnation. The pear also served as a source of polyphenols transferred to the plant material. Kopera and Mitek [15] used pear concentrate to enrich osmotically dehydrated pears in polyphenols. Yazidi et al. [16] used prickly pear molasses in the osmotic dehydration of oranges, achieving an increase in polyphenol content (as well as in ascorbic acid) compared to the fresh raw material. Another material that absorbed bioactive compounds was coconut. Bellary et al. [17] used curcumin, whereas Kawiji et al. [18] used ginger extract to introduce bioactive substances into coconut. Kowalska et al. [19] obtained sensorially attractive chips from Japanese quince by applying osmotic dehydration in a mixture of chokeberry juice and sucrose prior to convective drying. The concept of transferring extracted compounds was also used in patents. For example, Han and Sun [20] used a hyperosmotic multi-component solution containing, among other things, *Berberis jamesiana* fruit extract for the osmotic dehydration of blueberries.

Analysis of the available literature sources indicates that there is currently no data concerning the transfer of polyphenols from plants belonging to the genus *Agrimonia*. As for polyphenolic compounds from wild rose, Galus et al. [21] described the effect of dehydrating oranges in a mixture of wild rose juice and trehalose on the overall polyphenol content. Results of the analysis of apricots after vacuum impregnation in rose extract (Demir and Alpaslan [22]) are available—but they provide information about the content of volatile compounds. On the other hand, Stavropoulou and Giannakourou [23] used aqueous by-products from the *Rosa damascena* distillation process for the impregnation of mushroom pieces before osmotic dehydration; total polyphenols were determined. The products obtained appeared to maintain their quality better. A reduced total color change was noted, and reduced drip loss during frozen storage was observed compared to the untreated sample.

The analysis of polyphenol transfer is important due to their beneficial effects on the human body. They exhibit antioxidant activity, inhibit lipid peroxidation, scavenge free radicals, and have anticancer, anti-inflammatory, antimutagenic, and antidepressant properties. In particular, wild rose is considered a valuable source of polyphenolic compounds [24]. Additionally, the antimicrobial properties of rose extracts have been demonstrated, which may be significant for extending the shelf life of products containing them [25,26,27,28].

Plants from the genus *Agrimonia* are also rich sources of polyphenolic compounds with potential therapeutic properties [29,30]. Scientific studies have demonstrated that the specific mixture of phytocomponents from *Agrimonia* species exhibits α-glucosidase inhibitory [31], antioxidant, anti-inflammatory, and analgesic activities [32,33], stimulates the expression of proinflammatory cytokines in Caco-2 cells [34], and shows antibacterial and antibiofilm properties [35]. The presence of polyphenols in extracts from fragrant agrimony [29,30] may provide potential for the use of these extracts to enrich food products with polyphenols. Taking the above into account, it can be hypothesized that both plants constitute an interesting alternative for obtaining polyphenols for use in the process of osmotic dehydration.

The aim of the study was therefore to investigate the migration of polyphenols from fragrant agrimony (*Agrimonia procera* Wallr., *A. procera*) and the pseudo-fruits of rose (*Rosa rugosa*, *R. rugosa)*—both total polyphenols and their main representatives—during the osmotic dehydration of apples, which are one of the most popular fruit due to their health-promoting and nutritional properties [36].

## 2. Results and Discussion

### 2.1. Dry Matter Content, Water Loss and Solids Gain

**Figure 1:**
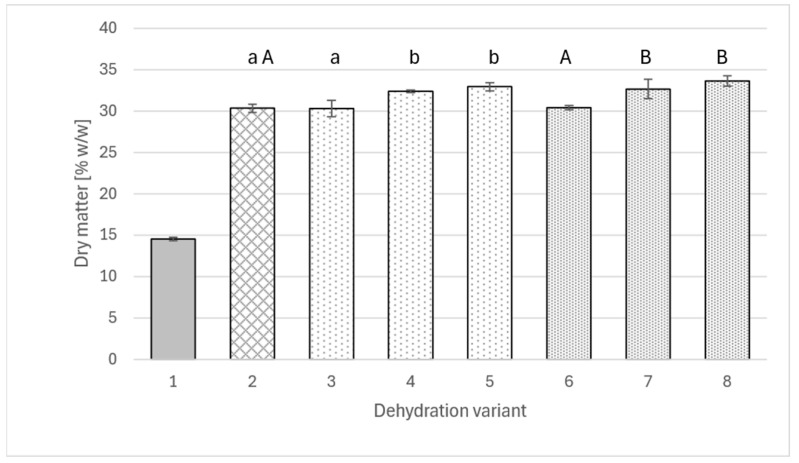
The content of dry matter in fresh apple (1), osmotically dehydrated (4 h; 40°C) in a 50% sucrose solution (2) and in sucrose solutions with the addition of rose extract (3–5) or agrimony (6–8); sucrose solutions with varying concentrations of total polyphenols: 2 g/L (3 and 6), 4 g/L (4 and 7), 6 g/L (5 and 8). Different lowercase letters indicate statistically significant differences (*p* ≤ 0.05) between values obtained for different contents of polyphenols from rose (from 0 to 6 g/L) (2, 3, 4, 5). Different uppercase letters indicate statistically significant differences (*p* ≤ 0.05) between values obtained for different contents of polyphenols from fragrant agrimony (from 0 to 6 g/L) (2, 6, 7, 8).

The data shown in Figure 1 indicate that an appropriately large addition of polyphenol extract to a sucrose solution (amounts used within the range applied in various studies [10,13,37,38] may have a beneficial effect on the dry matter concentration in dehydrated apples. Interestingly, in the case of both extracts (from rose and agrimony), the same relationship was observed, and the values were very similar.

While the use of solutions containing polyphenols at a concentration of 2 g/L did not yet cause a significant difference in the dry matter content of apples compared to the solution without the addition of extracts (for all, slightly over 30% *w*/*w*), increasing the extract addition to a level of 4 g of polyphenols per liter of solution resulted in a significant difference in dry matter concentration, reaching 32.4% *w*/*w* for rosehip extract and 32.7% *w*/*w* for fragrant agrimony extract, respectively. Increasing the addition of extracts to a level of 6 g of polyphenols per liter of solution did not produce another significant change, although in both cases, the dry matter contents were slightly higher than for the 4 g/L variant.

**Figure 2:**
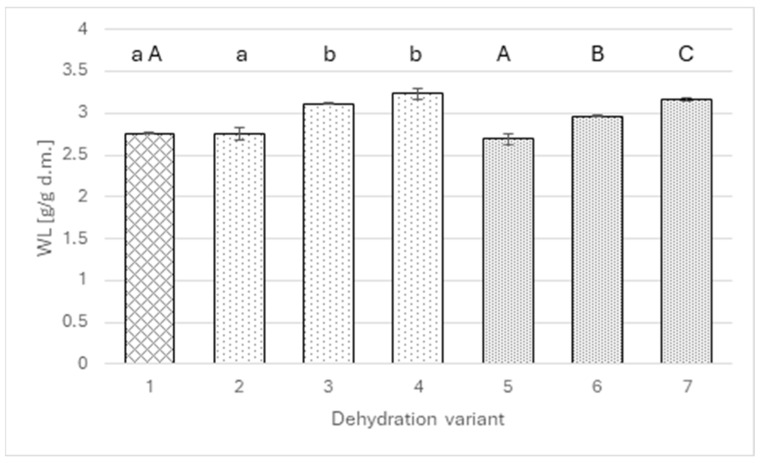
Water loss for osmotically dehydrated apple (4 h; 40°C) in a 50% sucrose solution (1) and in sucrose solutions with the addition of rose (2–4) or agrimony extract (5–7); sucrose solutions with different total polyphenol concentrations: 2 g/L (2 and 5), 4 g/L (3 and 6), 6 g/L (4 and 7). Different lowercase letters indicate statistically significant differences (*p* ≤ 0.05) between values obtained for different contents of polyphenols from rose (from 0 to 6 g/L) (1, 2, 3, 4). Different uppercase letters indicate statistically significant differences (*p* ≤ 0.05) between values obtained for different contents of polyphenols from fragrant agrimony (from 0 to 6 g/L) (1, 5, 6, 7).

The mass transfer analysis indicates that the factor responsible for the above differences is primarily water loss (Figure 2). A similar pattern of dependence occurs as in the case of dry matter. The addition of extracts up to a level of 2 g of polyphenols per liter of solution did not change the amount of water removed from the apples (approximately 2.7 g/g of dry matter), whereas increasing the polyphenol level to 4 g/L significantly raised water loss to 3.12 g/g DM in the wild rose variant and 2.96 g/g DM for agrimony. Interestingly, in the case of agrimony, another significant change was noted when the polyphenol level was increased to 6 g/L; in the case of wild rose, the statistical test did not show significance of the increase.

**Figure 3:**
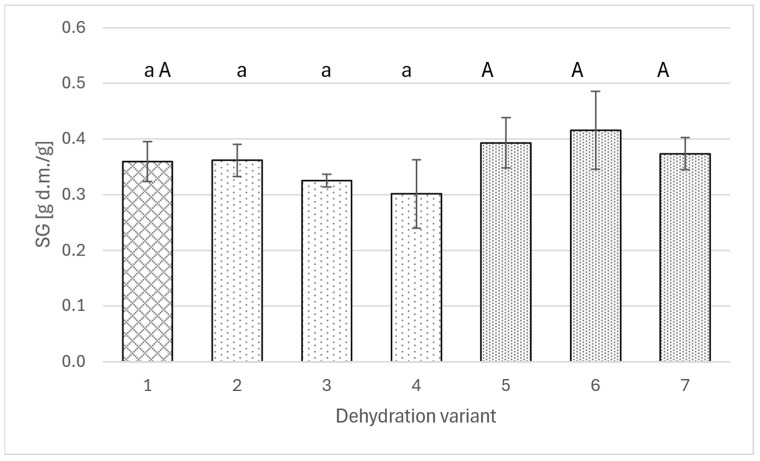
Solids gain for osmotically dehydrated apple (4 h; 40°C) in a 50% sucrose solution (1) and in sucrose solutions with the addition of rose (2–4) or agrimony extract (5–7); sucrose solutions with different concentrations of total polyphenols: 2 g/L (2 and 5), 4 g/L (3 and 6), 6 g/L (4 and 7). Different lowercase letters indicate statistically significant differences (*p* ≤ 0.05) between values obtained for different contents of polyphenols from rose (from 0 to 6 g/L) (1, 2, 3, 4). Different uppercase letters indicate statistically significant differences (*p* ≤ 0.05) between values obtained for different contents of polyphenols from fragrant agrimony (from 0 to 6 g/L) (1, 5, 6, 7).

On the other hand, the analysis of the transfer of the osmotic agent to the apple (Figure 3) does not allow for indicating a specific relationship between changes in solids gain and the amount of added extract. In no variant was a statistically significant difference obtained between the value achieved using the solution with polyphenols and the value for the pure sucrose solution. Solids gain ranged from 0.3 to 0.4 g DM/g.

A possible cause of more intensive dehydration of tissue in a hypertonic solution containing polyphenols is their interaction with the cell membrane lipids. According to Karonen [39], this is a complex issue, dependent on many factors, including the presence of specific polyphenols and their concentrations. The effect of polyphenols presence can be both positive, organizing the lipid layer, as well as negative, weakening the structure of the cell membrane, which in turn may be associated with its increased permeability. In general, in the case of the presence of components derived from fruit in hypertonic solutions, the importance of all substances of this origin on the course of osmoconcentration is indicated. In addition to polyphenols, sugars, acids, pectins, and mineral compounds also determine the process conditions by affecting the osmotic potential of the hypertonic solution, shaping its viscosity or acidity. The combined effects of these compounds can result in an increased efficiency of dehydration compared to a pure sucrose solution [40].

In the available literature, there are little data illustrating the differences in the course of osmotic dehydration in a solution of pure sucrose compared to a solution with the addition of a polyphenol extract (devoid of a significant portion of other fruit substances) by presenting the differences in the values of mass transfer indicators (water loss, solids gain) for a solution of pure sucrose and a solution containing polyphenols. Some data were presented by Samborska et al. [12]; they obtained a reduction in normalized solids gain (NSG) for apple after adding bilberry extract to the sucrose solution. In the case of a 5–15% (*w*/*w*) addition, NSG was lower by about 6–9% after 4 h of dehydration (value calculated based on data from the graph). At the same time, due to the way the data are presented, it is difficult to estimate to what extent the normalized water content changed after the addition of extracts to the sucrose solution. Ramos-Morales et al. [41], on the other hand, provide data on the dehydration of honeydew melon in the presence of hibiscus extract. In this case as well, the addition of the extract to the sucrose solution increased water loss (12.2–66.5 g water/100 g versus 6.50–57.6 g water/100 g). At the same time, the amount of osmotic substances migrating into the tissue decreased (0.35–7.43 g solids/100 g versus 1.93–15.7 g solids/100 g).

### 2.2. Polyphenol Composition of the Extracts

In the process of osmotic dehydration, extracts obtained from fragrant agrimony herb (*Agrimonia procera* Wallr.) and from the pseudo-fruit of *Rosa rugosa* were used, as described in Section 3.2. Both fragrant agrimony and wild rose belong to the Rosaceae family and are raw materials rich in tannins, including both hydrolysable tannins and condensed tannins. The hydrolysable tannins present in both fragrant agrimony and the pseudo-fruit of rose include ellagitannins, while the condensed tannins include proanthocyanidins. Furthermore, in both of these raw materials, the main ellagitannin is agrimoniin—an ellagitannin with a molecular mass of 1870, with a dimeric structure in which two α-galloyl-bis HHDP-glucose monomer units are connected by two gallic acid molecules via a GOG-type bond [42]. 

Table 1 presents the quantitative composition of polyphenols in the extract from *Agrimonia procera* Wallr. herb used in the osmotic dehydration of apples, whereas Table S1 in the Supplementary Materials provides the retention times, UV–Vis data, molecular weights, and MS/MS fragmentation ions of the polyphenols identified in the above-mentioned extract. Figure S1 in the Supplementary Materials shows the UV–Vis chromatograms of the *Agrimonia procera* Wallr. extract recorded at 250 and 360 nm. The presented data indicate that the extract from fragrant agrimony herb contains phenolic compounds belonging to the following groups: ellagitannins, phenolic acids, flavan-3-ols, flavones, and flavonols. The main groups of polyphenols in the studied extract were ellagitannins and flavan-3-ols (including polymeric proanthocyanidins), whose content in the extract was similar, amounting to 5200 mg/100 g, which accounted for about 35% of the total determined polyphenols. A characteristic ellagitannin of plants from the genus *Agrimonia* is the dimeric agrimoniin. In the extract from the fragrant agrimony herb, agrimoniin was present in an amount of 3966 mg/100 g and was the dominant polyphenolic compound (27% of the total polyphenols). The aforementioned polymeric proanthocyanidins were present in the extract in an amount of 4784 mg per 100 g. Ellagic acid and ellagic acid pentoside (both phenolic acids) together accounted for only slightly more than 0.2% of the extract’s mass. Noteworthy is the high content of flavones (2377 mg/100 g of extract) present in the form of glucosides and glucuronides of apigenin (contents of 16.5 and 1942 mg/100 g, respectively) and luteolin (contents of 104.5 and 313.9 mg/100 g, respectively). Particularly significant is the high content of apigenin and luteolin in the form of bioavailable glucuronides [43,44,45]. The group of flavonols identified in the analyzed extract includes derivatives of quercetin and kaempferol, with a total content of 1751 mg/100 g. The quercetin glycosides identified in the extract are quercetin arabinoglucoside (874 mg/100 g) and quercetin 3-O-rhamnosyl-7-O-glucoside and quercetin 3-O-galactoside (in very similar amounts, about 258 mg/100 g). In slightly smaller amounts, kaempferol derivatives were present in the extract, represented by kaempferol 3-O-rutinoside, kaempferol 3-O-glucoside, and tiliroside, with contents of 83.6, 44.1, and 212.3 mg/100 g of extract, respectively. The total content of polyphenols determined by the HPLC method was at the level of 14,773 mg/100 g, while the total polyphenol content determined by the spectrophotometric method using the Folin–Ciocalteu reagent was slightly lower, amounting to 12,260 mg/100 g of extract.

Table 2 summarizes the polyphenolic profile of the extract obtained from rose hip pomace and applied in the dehydration process. The corresponding analytical characteristics of the identified compounds—retention times, UV–Vis spectra, molecular weights, and MS/MS fragmentation ions—are provided in Table S2 of the Supplementary Materials. In addition, Figure S2 in the Supplementary Materials displays the UV–Vis chromatograms of the *Rosa rugosa* extract recorded at 250 and 360 nm.The extract contained ellagitannins at a level of 3048 mg/100 g. Main ellagitannin with a molecular mass of 1870 Da was tentatively identified as agrimoniin constituted 57.8% of the ellagitannins. Free ellagic acid, flavonols, including tiliroside, as well as quercetin and its hexoside (galactoside) were also identified. A significant portion of the phenolic compounds in the extract, 53.4%, were flavanols, with a content of 3812 mg/100 g, predominantly proanthocyanidins. The total polyphenol content determined by HPLC was at the level of 7143 mg/100 g, whereas the total polyphenol content determined spectrophotometrically using the Folin–Ciocalteu reagent was higher, reaching 11,382 mg/100 g of extract.

### 2.3. Transfer of Polyphenols During the Osmotic Dehydration of Apples

Rose and agrimony polyphenols contained in the osmotic solutions used in the presented study migrated into the apple, which resulted in a higher total polyphenol level in the products compared to the use of pure sucrose solutions. Table 3 presents the total polyphenol content determined spectrophotometrically using the Folin–Ciocalteu reagent in raw apples, apples dehydrated in sucrose solution, and apples dehydrated in sucrose solution enriched with extracts. The results are presented with reference to both the fresh and dry mass of apples intended for dehydration, as well as apples after the osmotic dehydration process. In apples intended for osmotic dehydration, the total polyphenol content was 108.5 mg GAE/100 g fresh matter (FM) and 746.8 mg GAE/100 g dry matter (DM), respectively. As a result of dehydration in a 50% sucrose solution, the total polyphenol content changed and amounted to 132.1 mg GAE/100 g FM and 435.5 mg GAE/100 g DM. The observed changes in total polyphenol content were associated both with the dehydration process occurring during drying and with the migration of polyphenols from the apple tissue into the osmotic solution. A statistically significant increase in polyphenol content compared to apples dehydrated in sucrose solution was observed for all doses used, with the type of extract being unimportant. Moreover, it was demonstrated that the applied dose of the extract had a statistically significant effect on the total polyphenol content in the dehydrated apples.

The dynamics of changes in polyphenol content during the dehydration of apples with the addition of polyphenol extracts from agrimony herb and rose pseudo-fruit, within the concentration range of 2–6 g/L of osmotic solution, were similar. It should be emphasized that the addition of polyphenols at a level of 4 g/L allowed the restoration of the polyphenol level (in relation to dry matter) observed in fresh apples, whereas a dose of 6 g/L caused a statistically significant increase in polyphenol content in dehydrated apples compared to fresh samples. When considering fresh apple mass, the addition of even just 2 g of polyphenols per liter of solution resulted in an increase in polyphenol content by 45–49%. The use of a dose of 6 g/L increased the level of polyphenols by more than 1.5 times.

In the further part of the study, an attempt was made to determine which polyphenolic compounds migrate into the fruit tissue and whether this process occurs with the same intensity for all compounds. Analysis of the polyphenol content in apples intended for osmotic dehydration, as well as in apples osmotically dehydrated in a sucrose solution and in a sucrose solution with the addition of extracts from fragrant agrimony herb or rose pseudo-fruit, performed using the HPLC method, enabled the assessment of the enrichment level of osmotically dehydrated apples with polyphenolic compounds characteristic of fragrant agrimony and rose.

Table 4 outlines the polyphenol content in fresh apples prepared for dehydration as well as in apples subjected to osmotic dehydration in a sucrose solution. Detailed analytical parameters of the detected polyphenols—retention times, UV–Vis characteristics, molecular weights, and MS/MS fragmentation ions—are listed in Table S3 in the Supplementary Materials. Figure S3 in the Supplementary Materials presents the UV–Vis chromatograms of ‘Champion’ apples (*Malus domestica* Borkh.) recorded at 250 and 360 nm. Table 5 and Table 6 show the polyphenol content in samples of apples osmotically dehydrated in a sucrose solution with the addition of polyphenol extract from fragrant agrimony and rose pseudo-fruit, respectively.

It was shown that fresh Champion apples (*Malus domestica* Borkh.) contained phenolic acids (chlorogenic acid and p-coumaric acid derivatives), quercetin glycoside, and flavanols, including polymeric proanthocyanidins, in a total amount of 417.3 mg/100 g DM. The osmotic dehydration process in a sucrose solution resulted in an almost twofold reduction in the concentration of phenolic acids, about a 1.5-fold decrease in the content of quercetin glycoside, and only a slight, approximately 10%, reduction in the amount of flavanols and proanthocyanidins. The observed decrease in the content of phenolic compounds after the osmotic dehydration process in a sucrose solution can be explained by their partial diffusion from the apple tissue into the osmotic solution due to a concentration gradient. Additionally, a certain degree of degradation of some compounds (phenolic acids, quercetin glycoside) is not excluded, which has already been observed during fruit dehydration [46]. The smaller decrease in the content of flavanols and proanthocyanidins may result from their limited extractability in the water-sugar environment and stronger binding to the cellular structures of the fruit resulting from the formation of proanthocyanidin–polysaccharide complexes within the cell wall matrix through hydrogen bonding and hydrophobic interactions [47].

In the case where dehydration was performed in a sucrose solution with the addition of fragrant agrimony extract, a decrease in chlorogenic acid concentration was observed along with an increase in extract concentration in the osmotic solution. In the case of rose extract, a decrease is noticeable for 4 and 6 g/L compared to 2 g/L.

The changes in the content of flavanols and proanthocyanidins looked quite different, as on the one hand they naturally occurred in apples (endogenous component), and on the other hand they were a significant polyphenolic component of the used extracts from fragrant agrimony and rose (exogenous component).

In apples dehydrated in a sucrose solution with the addition of 2 g of fragrant agrimony polyphenols per liter, the concentration of flavanols and proanthocyanidins was comparable to their concentration in fresh apples (flavanols and proanthocyanidins in fresh apples were 355 and 295 mg/100 g DM, respectively (Table 4), in apples dehydrated with the addition of 2 g per liter of fragrant agrimony, they were 324 and 282 mg/100 g DM, respectively (Table 5).

However, when the polyphenol level in the osmotic medium was increased, particularly to 6 g/L, a transfer of flavanols and proanthocyanidins from the osmotic solution to the fruit tissue was observed. Compared to fresh apples, the enrichment level of polymeric proanthocyanidins increased by 30% and 50%, respectively, for apples dehydrated in a sucrose solution containing fragrant agrimony polyphenols at doses of 4 and 6 g/L, respectively. In the case of flavanols, the enrichment level was slightly lower—20% and 30%, respectively. This indicates that polymeric proanthocyanidins migrated into the fruit tissue more intensively than monomeric free catechins.

Of particular importance is that the use of extract from fragrant agrimony herb as a component of the osmotic medium enabled the enrichment of apples with exogenous polyphenols such as ellagitannins, mainly agrimoniin, ellagic acid and its derivative, flavones represented by glycosides and glucuronides of apigenin and luteolin, as well as derivatives of quercetin and kaempferol. Moreover, the concentration of most of the mentioned polyphenolic compounds in dehydrated apples increased proportionally to the dose of extract used for dehydration.

In the case of dehydration in rose hip pseudo fruit extract at a dose of 2 g/L of polyphenols, the procyanidin content was slightly lower than in apples dehydrated in sacharose and amounted to 295 mg/100 g DM (Table 4) and 262 mg/100 g DM (Table 6), respectively. Increasing the polyphenol dose to 4 g/L caused a slight increase in content to 301 and 271 mg/100 g DM, respectively, and at a dose of 6 g/L, the content reached 352 and 320 mg/100 g DM. When using rose hip pseudo fruit extract, no increase in the content of flavonols and procyanidins was observed during dehydration, which occurred when using rapeseed extract. This may be related to the degree of polymerization of procyanidins DP = 6, which was higher than in fragrant agrimony extract DP = 4 (Table 1 and Table 2).

The use of extract from rose pseudo fruit for osmotic dehydration allowed the enrichment of apples with exogenous polyphenols contained in the extract, such as ellagitannins, mainly agrimoniin, ellagic acid, and flavonols, including quercetin hexoside (galactoside), quercetin, and tiliroside. Similarly, as in the case of using agrimony extract, the concentration of most of the aforementioned polyphenolic compounds in dehydrated apples increased with the increase in polyphenol dose.

In summary, it can be stated that the addition of polyphenol extracts from fragrant agrimony and rose pseudo fruit contributed to an increase in polyphenol content in dehydrated apple tissue, surpassing the initial level in fresh apples. The use of these extracts also allowed for the enrichment of apples with exogenous polyphenolic compounds from the osmotic solution. Considering numerous literature studies on the beneficial properties of extracts from agrimony and rose [25,26,27,28,48], their addition to the osmotic dehydration of apples seems justified, and the resulting final product may exhibit a higher health beneficial potential than the raw material.

The obtained results can be compared to data from the literature. However, the experiments described in the publications were conducted under different configurations, that is, using, for example, different fruit, different solutions, or different osmoconcentration conditions.

Among the few publications describing the use of wild rose is the one by Galus et al. [21], which described the effect of dehydrating oranges in a 50% solution (30 °C, fruit-to-syrup ratio 1:4) prepared from wild rose juice and trehalose (the polyphenol content was not specified). The total polyphenol content in oranges (expressed as gallic acid equivalents—GAE) was obtained at a level of 1929 g/100 g dry matter. This result is more than twice as high compared to the maximum values reported in this publication (Table 3).

Stavropoulou & Giannakourou [23], after applying aqueous by-products from the *Rosa damascena* distillation process (polyphenols 1539.14 mg/L; impregnation conditions: 10 min, followed by the addition of glycerol and NaCl, 40 °C), obtained mushrooms containing approximately 730 mg GAE/100 g after 130 min of osmoconcentration (data from the graph). As the authors report, after this period the moisture content reached about 50% (48.25% for the variant without the initial 10-min impregnation), from which it can be inferred that the level of polyphenols per 100 g DM is about 1400 mg. This result is approximately 1.7 times higher compared to the results of our study (Table 3) for a dose of 6 g/L, although it was obtained using more than four times the amount of hypertonic solution (1:15).

As for the osmoconcentration of apples, Cichowska et al. [11] obtained a level of 590 mg GAE/100 g DM after osmoconcentration in a 60 °Bx mixture (1:1) of sucrose and concentrated chokeberry juice (polyphenol content not provided; conditions: 120 min, 40 °C, fruit/solution 1:4). The concentrations obtained in our experiments are 1.4 times higher when using a dose of 6 g/L and 1.2 to 1.3 times higher for a dose of 4 g/L.

Hernández-Carranza et al. [10] used hibiscus extract (20% *w*/*v* extract standardized to 50 °Bx, fruit/solution 1:5; total polyphenol content of extract 423.8 ± 1.9 mg GAE/100 g). After four hours of osmoconcentration at temperatures of 30–50 °C, the total polyphenol content reached approximately 55–62 mg/100 g (read from the graph). In our experiments, the total polyphenol content ranged from 157 to 273 mg/100 g (depending on the dose) for the wild rose extract and from 162 to 278 mg/100 g for the agrimony extract (Table 3); thus, it was almost 4.5 times higher (for a dose of 6 g/L) than the values reported in the aforementioned literature source.

Masztalerz et al. [49] performed osmotic dehydration of apple slices in 40 °Bx chokeberry juice (polyphenol content not reported; conditions: 40 °C, apples/solution 1/3, 120 min, additionally assisted by ultrasound/vacuum/microwave). The highest obtained total polyphenol content was 217 mg GAE/100 g DM in the case of osmoconcentration assisted by ultrasound. In the case of exposure only to the concentrated solution, the maximum level of polyphenols was 175 mg GAE/100 g DM. These are therefore lower values than those presented in this article (for the lowest dose of polyphenols from agrimony and rose −2 g/L −518 and 531 mg/100 g DM, respectively, Table 3).

In addition to presenting the total polyphenol content, one can also encounter information regarding the levels of selected polyphenolic compounds. Castagnini et al. [13] reported the contents of selected anthocyanins after vacuum impregnation of apples in blueberry juice. Delphinidin, cyanidin, and malvidin were present in amounts of 3.19, 3.46, and 6.04 mg/100 g DM, respectively (determined after lyophilization)—a total of 12.69 mg/100 g DM. This effect was achieved with the content of these three anthocyanins in the juice at the level of 67.62 mg/L. Anthocyanins were not detected in our extracts.

There are limited data in the literature regarding the relationship between the level of polyphenolic compounds in fruits after osmoconcentration in solutions with different polyphenol contents from plant extracts, while maintaining the same concentration of the hypertonic solution. Some data on anthocyanin content were presented by Channanavar et al. [8], although they concern a product that, after osmoconcentration, was also subjected to convective drying. However, a significant influence (as observed in the studies presented in this article) of the initial content of the above-mentioned polyphenolic compounds in the hypertonic solution on the level of these compounds in pineapple is evident. Increasing the proportion of kokum extract (60 °Bx; anthocyanin content not provided) in the hypertonic solution (60 °Bx) from 50% to 100% raised the anthocyanin level from 2.65 mg/100 g to 4.34 mg/100 g, which is an increase of 64%.

## 3. Materials and Methods

### 3.1. Materials

The study used Champion apples (Champion apples are one of the most popular cultivars in Poland), fragrant agrimony herb *Agrimonia procera* Wallr. and pomace from *Rosa rugosa* pseudo-fruit. The apples were purchased at a local supermarket, the fragrant agrimony and rose came from field cultivation near the city of Łódź. The substances used were: sucrose (Pfeifer & Langen Polska S.A., Glinojeck, Poland), agrimoniin (standard for ET, obtained at the Institute of Food Technology and Analysis), ellagic acid, chlorogenic acid, p-coumaric acid, quercetin-3-O-glucoside, quercetin-3-O-rhamnoside, quercetin 3-O-rhamnosyl-7-O-glucoside, quercetin-3-O-galactoside, apigenin-7-O-glucoside, apigenin-7-O-glucuronide, luteolin-7-O-glucoside, luteolin-7-O-glucuronide, kaempferol-3-O-β-d-(6″-E-p-coumaryl)-glucopyranoside, kaempferol-3-O-glucoside (Extrasynthese, Genay, France).

### 3.2. The Method of Obtaining Extracts

The extracts were prepared from frozen pomace of rose pseudo-fruit and dried herb of fragrant agrimony. The method was previously described in our earlier research [23,24,25,28]. The material was ground and then poured with a mixture of acetone:water:formic acid (25:24.95:0.05, *v*/*v*/*v*) at a ratio of 1:4 (*w*/*v*) for rose pseudo-fruit and 1:20 (*w*/*v*) for fragrant agrimony herb. Extractions were carried out at room temperature for 1 h using a DOS-10L orbital shaker (ELMI SIA, Riga, Latvia) at 130 rpm, and then statically for 23 h. After this time, the extracts were decanted, and the post-extraction residue was subjected to another extraction performed in a similar manner. Extracts from the first and second stages were combined, filtered (using an HOBRA S40 N filtration barrier (HOBRA—Školnik s.r.o. Broumov, Czech Republic)), and then concentrated using a Heidolph 24/7 automatic evaporator (Heidolph Instruments GmbH & Co. KG, Schwabach, Germany). Next, the extract was lyophilized (0.18 mbar, −36 °C, 48 h) in a Christ Alpha 1–2 LD plus (Martin Christ Gefriertrocknungsanlagen GmbH, Osterode am Harz, Germany) laboratory freeze dryer. In the extracts, after their dissolution in 2 mL of methanol:water:formic acid (70:29.9:0.1, *v*/*v*/*v*), the content of polyphenolic compounds was determined according to the procedure described in the following Section 3.7, Section 3.8 and Section 3.9. Until the experiments were performed, the freeze-dried extracts were stored in tightly sealed polyethylene containers at a temperature of −18 °C.

### 3.3. Osmotic Dehydration

In the experiments, a 50% sucrose solution was used, into which an extract from fragrant agrimony or wild rose was introduced to achieve a total polyphenol concentration of 2, 4, or 6 g/L.

The apples were cut into cubes with an edge length of approximately 0.7 cm. About 25 ± 1 g of the sample was weighed into screw-cap containers, and four times as much hypertonic solution was added. The containers were placed in a water bath with a shaker (GFL 1086, Lauda GFL, Burgwedel, Germany) set to a temperature of 40 °C and a shaking frequency of 170 cycles/min. The dehydration was carried out for four hours. After dehydration, the apple was transferred onto a sieve, which was dipped twice in distilled water. The apple was then dried on filter paper and frozen in polyethylene resealable bags.

### 3.4. Dry Matter Content

Samples of approximately 3 g were weighed into weighing vessels. Next, they were dried at 70 ± 2 °C for 24 h under reduced pressure (VO 400, Memmert, Büchenbach, Germany). The samples were then cooled in a desiccator and weighed. The dry matter content (DM) was calculated according to the formula:

DM = 100 × mk/m0   [%]

m0—weight of sample before drying [g]
mk—weight of sample after drying [g]

### 3.5. Water Loss and Solid Gain Calculation

The following formulas were used to calculate water loss (WL) and solids gain (SG):

WL = [m0 (1 − s0) − mk (1 − sk)]/m0s0 [g H_2_O/g initial dry matter]

SG = (mksk − m0s0)/m0s0 [g dry matter/g initial dry matter]

where

m0—weight of sample before osmotic dehydration [g]
mk—weight of sample after osmotic dehydration [g]
s0—solids content before osmotic dehydration [g of dry matter per g]
sk—solids content after osmotic dehydration [g of dry matter per g]

### 3.6. Phenolic Extraction

1 g of ground sample was extracted with 4 mL of solvent (methanol:formic acid 99.9/0.1 (*v*/*v*)). The samples were vortexed, sonicated for 15 min and left overnight in the dark, and then centrifuged at 10,000× *g*. The extract was poured into a volumetric flask, and the residues were re-extracted twice with 4 mL and 3 mL of solvent (acetone/water/formic acid 70/29.9/0.1 (*v*/*v*/*v*)). The final extracts were combined and made up to 10 mL in a volumetric flask. The resulting extracts were subjected to HPLC analysis according to the procedure described in Section 3.7.

### 3.7. Identification and Quantification Phenolic Compounds by UHPLC-DAD-MS

UHPLC Ultimate 3000 chromatographic system (Thermo Fisher Scientific, Germering, Germany) equipped with a DAD detector and a Q Exactive Orbitrap mass detector was used for phenolic compounds identification and quantification. The separation was performed using a Luna Omega 1.6 μm C18 100Å column 150 × 2.1 mm (Phenonomenex, Torrance, CA, USA). Phase A was 0.5% (*v*/*v*) formic acid in water, while phase B was mixture of acetonitrile: methanol:water, formic acid (63:20:16.5:0.5 *v*/*v*/*v*/*v*). The following gradient was applied: 0–2 min, 5% B; 2–12 min, 5–28% B; 12–20 min, 28–73% B; 20–25 min, 73% B; 25–27 min, 73–5% B; 27–35 min, 5% B. The column temperature was 40 °C, the flow rate 0.4 mL/min, and injection volume was 5 μL. The mass detector parameters were as follows: negative ionization mode, the evaporator temperature was 400 °C, electrospray voltage of 4 kV, and a spray capillary temperature was 380 °C. Nitrogen drying and auxiliary gas flow rates were 60 and 15 units, respectively. Data were collected in the range of 150–2000 *m*/*z* in full MS and data-dependent MS2 modes. UV-Vis detection was performed in the range of 200–600 nm, with a wavelength of 250 nm selected for the quantitative analysis of ellagitannins, 320 nm for derivatives of chlorogenic and p-coumaric acid, and 360 nm for flavonols and flavones. The quantification of identified compounds was based on standard curves derived from available standards (agrimoniin (for ET), ellagic acid, chlorogenic acid, p-coumaric acid, quercetin-3-O- glucoside, quercetin-3-O- rhamnoside, quercetin-3-O-galactoside, apigenin-7-O-glucoside, apigenin-7-O-glucuronide, luteolin-7-O-glucoside, luteolin-7-O-glucuronide, kaemferol-3-O-β-d-(6″-E-p-coumaryl)-glucopyranoside, kaempferol-3-O-glucoside.). Compounds for which no reference standards were available were quantified as equivalents of the standards with the most similar chemical structure. All samples were analyzed in four replicates.

### 3.8. Flavanols: Proanthocyanidins and Free Catechins

The content of proanthocyanidins was determined using the method described by Sójka et al. [50]. For the analysis, 5 mg of the lyophilized powdered extracts and 100 mg of sample after dehydration (a weighed sample of apples was lyophilized before analysis) was weighed into 2 mL plastic test tubes. The phloroglucinolysis reaction procedure was performed at 50 °C for 30 min. Free catechins were determined from the solutions prepared for UHPLC-MS analysis. Separation of the phloroglucinolysis products, including catechin adducts with phloroglucinol, released catechins, and free catechins, was performed using a Shimadzu chromatograph (Shimadzu Corporation, Tokyo, Japan), equipped with an LC-20AD pump, SIL-20ASHT autosampler, CTO-10ASVP thermostat, and RF-10AXL detector. A Gemini 5u C18 110Å column (Phenomenex, Torrance, CA, USA) with dimensions of 250 mm × 4.6 mm and a 5 μm particle size was used. All samples were analyzed in four replicates.

### 3.9. Phenolic Compounds Spectrophotometric Method Folin-Ciocalteu

Total phenolics compounds were measured using the method described by Singleton and Rossi [51] with some modifications. Briefly, to the 25 mL flask 0.5 mL of sample, 0.25 mL Folin-Ciocalteu reagent, and 2.5 mL 20% Na_2_CO_3_ were added and filled with water up to 25 mL. Incubation was conducted for 1 h at room temperature. Absorbance was measured at 720 nm. A The results were expressed as mg gallic acid equivalents per 100 g of lyophilized extract or 100 g apple sample after dehydration. All samples were analyzed in four replicates.

### 3.10. Experimental Design and Statistical Analysis

The experiments were performed in two technological repetitions. All analytical tests were conducted in 3 analytical repetitions. One-way Anova with a Duncan test was used (Statistica 12, Statsoft, Kraków, Poland). The results were considered statistically significant at *p* ≤ 0.05.

## 4. Conclusions

The addition of polyphenol extract from fragrant agrimony or rose to a hypertonic solution may increase the dry matter concentration in dehydrated apples compared to a pure sucrose solution. The main reason is the more intensive water outflow from the fruit tissue; the transfer of sucrose into the tissue may remain unchanged. During osmotic dehydration in a pure sucrose solution, there is a decline in apple-native polyphenols, especially those not bound to cell walls, such as chlorogenic acid, p-coumaric acid, and quercetin glycosides. Osmotic dehydration of apples in sucrose solutions with the addition of fragrant agrimony and rose extracts increases the polyphenol content in the dehydrated apple and also allows the final product to be enriched with polyphenolic compounds not naturally present in the raw material, such as ellagitannins, ellagic acid, flavonols, or flavones.

The use of fragrant agrimony herb extract into the osmotic medium enabled the enrichment of apples with exogenous polyphenols, including ellagitannins—primarily agrimoniin—ellagic acid and its derivatives, flavones represented by apigenin and luteolin glycosides and glucuronides, as well as quercetin and kaempferol derivatives. The use of extract from rose pseudo fruit for osmotic dehydration allowed the enrichment of apples with exogenous polyphenols contained in the extract. Alongside ellagitannins, the apples absorbed a range of flavonols, including quercetin derivatives such as quercetin galactoside, quercetin, and tiliroside. To comprehensively validate the use of fragrant agrimony and rosehip extracts in the dehydration of apples as a means of enhancing their polyphenolic profile, it is essential to incorporate investigations assessing the process-induced alterations in color parameters, textural characteristics, and overall sensory attributes of the resulting product.

## Figures and Tables

**Table 1:** Polyphenolic profile of the extract from fragrant agrimony (*Agrimonia procera* Wallr.) herb (mg/100 g DM).

Compound	Concentration of Polyphenols (mg/100 g DM)
Agrimoniin	3966 ± 116
Sum of ellagitannins	5217 ±157
Ellagic acid	169.3 ± 7.2
Ellagic acid pentoside	58.6 ± 2.3
Apigenin 7-O-glucoside	16.5 ± 0.1
Apigenin 7-O-glucuronide	1942 ± 45
Luteolin 7-O-glucoside	104.5 ± 4.1
Luteolin 7-O-glucuronide	313.9 ± 4.2
Quercetin arabinoglycoside	874.0 ±18.9
Quercetin 3-O-ramnozyl-7-O-glucoside	257.8 ± 4.4
Quercetin 3-O-galactoside	258.1 ± 12.6
Kaempferol 3-O-rutoside	83.6 ± 1.7
Kaempferol 3-O-glucoside	44.1 ± 0.7
KpCG *	212.3 ± 4.7
Proanthocyanidins **	4784 ± 167
Sum of flavanols	5200 ± 169
DP	4.1 ± 0.1
Total polyphenols HPLC ***	14,773 ± 347
Total polyphenols F-C ****	12,260 ± 179

The values are presented as the mean ± standard deviation in mg/100 g DM; KpCG *—kaempferol-3-O-β-d-(6″-E-p-coumaroyl)-glucopyranoside (tiliroside); ** Proanthocyanidins are part of flavanols group; DP- degree of flavanols polymerization; Total polyphenols HPLC ***—the total content of individual polyphenols determined by the HPLC method (sum of ellagitannins, phenolic acids, flavones, flavonols, and flavanols); Total polyphenols F-C ****—total polyphenol content determined by the spectrophotometric method using the Folin-Ciocalteu reagent in mg GAE/100 g.

**Table 2:** Polyphenolic profile of the extract from rosehip pomace (mg/100 g DM).

Compound	Concentration of Polyphenols (mg/100 g DM)
Agrimoniin	1763 ± 43
Sum of ellagitannins	3049 ± 82
Ellagic acid	162.9 ± 5.3
Quercetin-3-O-galactoside	18.5 ± 1.4
Quercetin	32.1 ± 1.6
KpCG *	68.7 ± 2.0
Proanthocyanidins **	3733 ± 144
Sum of flavanols	3812 ± 142
DP	6.2 ± 0.1
Total polyphenols HPLC ***	7143 ± 97
Total polyphenols F-C ****	11,382 ± 415

The values are presented as the mean ± standard deviation in mg/100 g DM; KpCG *—kaempferol-3-O-β-d-(6″-E-p-coumaroyl)-glucopyranoside (tiliroside); **—proanthocyanidins are part of flavanols group; DP- degree of flavanols polymerization; Total polyphenols HPLC ***—the total content of individual polyphenols determined by the HPLC method (sum of ellagitannins, ellagic acid, quercetin galactoside, KpCG and flavanols); Total polyphenols F-C ****—total polyphenol content determined by the spectrophotometric method using the Folin-Ciocalteu reagent in mg GAE/100 g.

**Table 3:** Total polyphenol content of fresh and osmotically dehydrated apples determined by the Folin–Ciocalteu spectrophotometric method (mg GAE/100 g FM and mg GAE/100 g DM).

	Total Polyphenol Content	
Sample	mg GAE/100 g FM	mg GAE/100 g DM
Fresh apple	108.5 ± 7.4 ab	746.8 ± 59.1 c
OD apples in 50% sucrose	132.0 ± 6.6 b	435.5 ± 23.3 a
OD apples in 50% sucrose + 2 g/L PPs RH	156.9 ± 9.4 c	517.8 ± 31.9 b
OD apples in 50% sucrose + 4 g/L PPs RH	224.4 ± 9.0 d	693.5 ± 28.4 c
OD apples in 50% sucrose + 6 g/L PPs RH	272.5 ± 9.7 f	827.1 ± 22.4 d
OD apples in 50% sucrose + 2 g/L PPs AP	161.5 ± 12.6 c	530.7 ± 39.7 b
OD apples in 50% sucrose + 4 g/L PPs AP	241.9 ± 6.2 e	741.2 ± 40.0 c
OD apples in 50% sucrose + 6 g/L PPs AP	277.7 ± 8.0 f	825.0 ± 18.0 d

The values are presented as the mean ± standard deviation in mg/100 g FW and mg/100 g DM; within the same column, means with different letters are significantly different at *p* ≤ 0.05; GAE—gallic acid equivalents; FM—fresh matter; DM—dry matter; OD apples in 50% sucrose—osmotically dehydrated apples in 50% sucrose solution; PPs RH—polyphenols from rose hip extract; PPs AP—polyphenols from *Agrimonia procera* Wallr. extract.

**Table 4:** HPLC-determined content of individual polyphenolic compounds in fresh and osmotically dehydrated apples.

Compound	Fresh Apple	OD Apples in 50% Sucrose
	mg/100 g DM	
Chlorogenic acid	53.6 ± 8.5 a	29.0 ± 9.6 b
p-Coumaric acid	6.7 ± 0.2 a	3.7 ± 0.3 b
Quercetin rhamnoside	1.6 ± 0.2 a	1.1 ± 0.2 b
Proanthocyanidins	295.5 ± 28.6	264.1 ± 16.3
Sum of flavanols	355.4 ± 25.2 a	313.7 ± 19.7 b
Total polyphenols HPLC	417.3 ± 16.4 a	347.5 ± 28.1 b

The values are presented as the mean ± standard deviation in mg/100 g DM; within the same row, means with different letters are significantly different at *p* ≤ 0.05; DM—dry matter; OD apples in 50% sucrose—osmotically dehydrated (4 h; 40 °C) apples in 50% sucrose solution.

**Table 5:** HPLC-determined polyphenolic composition of osmotically dehydrated apples in sucrose solution with the addition of polyphenolic extract from fragrant agrimony *Agrimonia procera* Wallr. (mg/100 g DM).

Compound	Concentrations of Polyphenols in OD Apples in 50% Sucrose +		
	2 g/L PPs AP	4 g/L PPs AP	6 g/L PPs AP
	mg/100 g DM		
Chlorogenic acid *	23.7 ± 0.8 a	20.5 ± 0.4 b	14.7 ± 2.2 c
*p*-Coumaric acid *	3.0 ± 0.1 a	2.6 ± 0.3 b	2.6 ± 0.2 b
Quercetin rhamnoside *	0.64 ± 0,05 a	0.52 ± 0.10 b	0.38 ± 0.04 c
Proanthocyanidins **	282.8 ± 16.2 b	387.7 ± 26.6 a	437.3 ± 55.1 a
Sum of flavanols	324.0 ± 17.0 b	431.4 ± 28.2 a	470.5 ± 38.8 a
Agrimoniin	67.5 ± 0.5 c	126.7 ± 2.6 b	195.8 ± 5.2 a
Sum of ETs	108.2 ± 1.1 c	179.8 ± 1.9 b	266.9 ± 7.5 a
Ellagic acid	6.0 ± 0.1 c	8.0 ± 0.1 b	10,9 ± 0.30 a
Ellagic acid pentoside	3.6 ± 0.1 b	3.7 ± 0.2 b	4.1 ± 0.1 a
Apigenin 7-O-glucoside	1.4 ± 0.1	1.3 ± 0.1	1.3 ± 0.08
Apigenin 7-O-glucuronide	25.3 ± 0.4 c	54.2 ± 1.9 b	86.1 ± 2.3 a
Luteolin 7-O-glucoside	1.4 ± 0.1 c	2.9 ± 0.1 b	4.5 ± 0.3 a
Luteolin 7-O-glucuronide	6.6 ± 0.1 c	10.5 ± 0.2 b	15.1 ± 0.4 a
Quercetin arabinoglycoside	12.3 ± 0.2 c	25.1 ± 0.8 b	39.6 ± 1.2 a
Quercetin 3-O-rhamnosyl-7-O-glucoside	4.2 ± 0.1 c	7.4 ± 0.4 b	11.5 ± 0.2 a
Quercetin 3-O-galactoside	4.5 ± 0.1 c	7.7 ± 0.3 b	11.5 ± 0.6 a
Kaempferol 3-O-rutoside	1.4 ± 0.1 c	2.5± 0.1 b	3.7 ± 0.1 a
Kaempferol 3-O-glucoside	0.89 ± 0.05 c	1.7 ± 0.1 b	2.6 ± 0.1 a
KpCG ***	4.9 ± 0.1 c	8.3 ± 0.1 b	11.8 ± 0.4 a
Total polyphenols HPLC	531.7 ± 17.7 c	768.1 ± 25.1 b	958.5 ± 26.4 a

The values are presented as the mean ± standard deviation in mg/100 g DM; within the same row, means with different letters are significantly different at *p* ≤ 0.05; DM—dry matter; OD apples in 50% sucrose—osmotically dehydrated (4 h; 40 °C) apples in 50% sucrose solution; *—endogenous polyphenols in apple; **—proanthocyanidins are part of flavanols group; KpCG ***—kaempferol-3-O-β-d-(6″-E-p-coumaroyl)-glucopyranoside (tiliroside); PPs AP—polyphenols from *Agrimonia procera* Wallr. extract.

**Table 6:** HPLC-determined polyphenolic composition of osmotically dehydrated apples in sucrose solutions with the addition of rosehip extract (mg/100 g DM).

Compound	Concentrations of Polyphenols in OD Apples in 50% Sucrose +		
	2 g/L PPs RH	4 g/L PPs RH	6 g/L PPs RH
	mg/100 g DM		
Chlorogenic acid *	22.4 ± 0.9 a	15.3 ± 0.5 b	16.1 ± 0.8 b
p-Coumaric acid *	2.9 ± 0.1 a	2.5 ± 0.0 b	2.8 ± 0.1 a
Quercetin rhamnoside *	0.75 ± 0.10 a	0.50 ± 0.02 b	0.53 ± 0.0 b
Proanthocyanidins **	262.2 ± 17.6 b	270.8 ± 26.3 b	320.3 ± 2.4 a
Sum of flavanols	296.0 ± 19.4 b	300.8 ± 26.0 b	352.4 ± 2.3 a
Agrimoniin	32.0 ± 0.9 c	57.2 ± 1.5 b	81.3 ± 2.6 a
Sum of ETs	82.1 ± 2.2 c	122.7 ± 1.5 b	169.8 ± 4.3 a
Ellagic acid	5.5 ± 0.1 c	7.3 ± 0.1 b	9.7 ± 0.4 a
Quercetin	1.7 ± 0.0 c	2.1 ± 0.0 b	2.8 ± 0.1 a
KpCG ***	1.8 ± 0.1 c	2.7 ± 0.1 b	3.6 ± 0.3 a
Total polyphenols	413.1 ± 15.2 c	454.1 ± 26.8 b	557.6 ± 6.3 a

The values are presented as the mean ± standard deviation in mg/100 g DM; within the same row, means with different letters are significantly different at *p* ≤ 0.05; DM—dry matter; OD apples in 50% sucrose—osmotically dehydrated (4 h; 40 °C) apples in 50% sucrose solution; *—endogenous polyphenols in apple; **—proanthocyanidins are part of flavanols group; KpCG ***—kaempferol-3-O-β-d-(6″-E-p-coumaroyl)-glucopyranoside (tiliroside); PPs RH—polyphenols from rose hip extract.

## Data Availability

The data presented in this study are available on request from the corresponding author.

## Supplementary Materials

The following supporting information can be downloaded at: https://www.mdpi.com/article/10.3390/molecules30244708/s1, Table S1: LC-MS identification of polyphenolic compounds in extracts from *Agrimonia procera* Wallr., Table S2: LC-MS identification of polyphenolic compounds in extracts from *Rosa rugosa*, Table S3: LC-MS identification of polyphenolic compounds in Champion apples (*Malus domestica* Borkh.), Figure S1: UV–Vis chromatograms of *Agrimonia procera* Wallr. extract acquired at 250 and 360 nm, Figure S2. UV–Vis chromatograms of Rosa rugosa extract acquired at 250 and 360 nm. Figure S3: UV–Vis chromatograms of Champion’ apples (*Malus domestica* Borkh.) acquired at 250 and 360 nm.
