# Supplementary material for: Osmotic Dehydration of Apples in a Saccharose Solution Containing Fragrant Agrimony or Rosehip Extract
Source: Molecules. 2025 Dec 9;30(24):4708. doi: 10.3390/molecules30244708 (PMC12735750; doi:10.3390/molecules30244708)
Supplement: Supplementary file 1 [file molecules-30-04708-s001.zip › molecules-4011135-supplementary.pdf]

Table 1. LC-MS identification of polyphenolic compounds in extracts from *Agrimonia procera* Wallr.

Peak no.	Compound	RT [min.]	UV-Vis λ_{max} [nm]	Precursor ion (*m/z*)		MS/MS fragment ions (*m/z*)
				$[M-H]^-$	$[M-2H]^{2-}$	
1	Quercetin arabinoglycoside	13.96	256, 355	595.13263		445.08, **300.03**
2	Ellagic acid pentoside	14.35	228, 351	433.04267		312.06, **300.99**
3	Agrimoniin	14.43	229sh		934.07497	1567.12, 1265.12, 1085.08, 935.07, 783.06, 633.07, 481.06, **301.00**
4	Quercetin rhamnoglucoside	14.70	255, 351	609.14799		343.05, **301.03**, 179.00, 151.00
5	Ellagic acid	14.86	253, 367	300.99967		-
6	Quercetin 3-*O*-galactoside	15.00	267, 352	463.08944		**301.04**
7	Luteolin glucoside	15.20	257, 266, 357	447.09480		**285.04**, 179.00, 151.00
8	Luteolin 7-O-glucuronide	15.62	255, 266, 349	461.07396		357.06, 327.05, **285.04**, 175.02, 113.02
9	Keampferol 3-O-rutinoside	15.94	267, 347	593,15283		429.18, 327.06, **285.04**, 179.06
10	Keampferol 3-O-glucoside	16.37	267, 347	447,09430		**285,04,** 269,05, 175,02, 151,00, 113,03
11	Apigenin 7-O-glucoside	16.50	268, 335	431.10007		**269.04,** 175.01, 151.00
12	Apigenin 7-O-glucuronide isomer 2	16.57	267, 338	445.07087		**269.05**, 175.02, 113.02
13	KpCG*	18.73	267, 314	593.13159		447.09, **285.04**

RT - retention time; λmax – maximum absorbance; MS – the first mass spectrum (pseudomolecular ions); MS/MS – the second mass spectrum (fragment ions); KpCG* - kaempferol-3-O-β-d-(6″-E-p-coumaroyl)-glucopyranoside (tiliroside).

Table 2. LC-MS identification of polyphenolic compounds in extracts from *Rosa rugosa*

Peak no.	Compound	RT [min.]	UV-Vis λ_{max} [nm]	Precursor ion (*m/z*)		MS/MS fragment ions (*m/z*)
				$[M-H]^-$	$[M-2H]^{2-}$	
1	Agrimoniin	14.38	231		934.08297	1567.16, 1265.15, 1085.08, 935.09, 783.07, 633.07, 481.06, **301.00**
2	Ellagic acid	14.78	253, 367	300.9996		-
3	Quercetin 3-*O*-galactoside	15.10	267, 352	463.0894		**301.04**
4	Quercetin	18.60	240, 362	301.0388		**-**
5	KpCG*	18.74	267, 315	593.1362		447.09, **285.04**

RT - retention time; λmax – maximum absorbance; MS/MS – the second mass spectrum (fragment ions); KpCG* - kaempferol-3-O-β-d-(6″-E-p-coumaroyl)-glucopyranoside (tiliroside).

Table 3. LC-MS identification of polyphenolic compounds in Champion apples (*Malus domestica* Borkh.)

Peak no.	Compound	RT [min.]	UV-Vis λ_{max} [nm]	Precursor ion (*m/z*) $[M-H]^-$	MS/MS fragment ions (*m/z*)
1	Chlorogenic acid	9.46	227, 326	353.08913	191, 179, **173,** 135
2	p-Coumaric acid	10.93	312	162.8401	-
3	Quercetin rhamnoside	16.37	255, 353	447.0993	**301.03**

RT - retention time; λmax – maximum absorbance; MS/MS – the second mass spectrum (fragment ions).

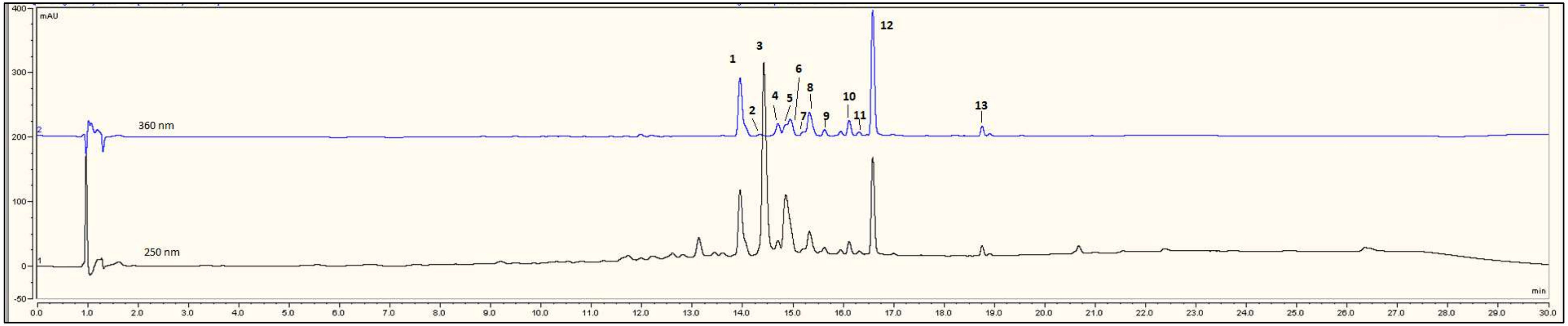

Figure 1. UV–Vis chromatograms of Agrimonia procera Wallr. extract acquired at 250 and 360 nm; Peak numbers correspond to table 1 in Supplementary material.

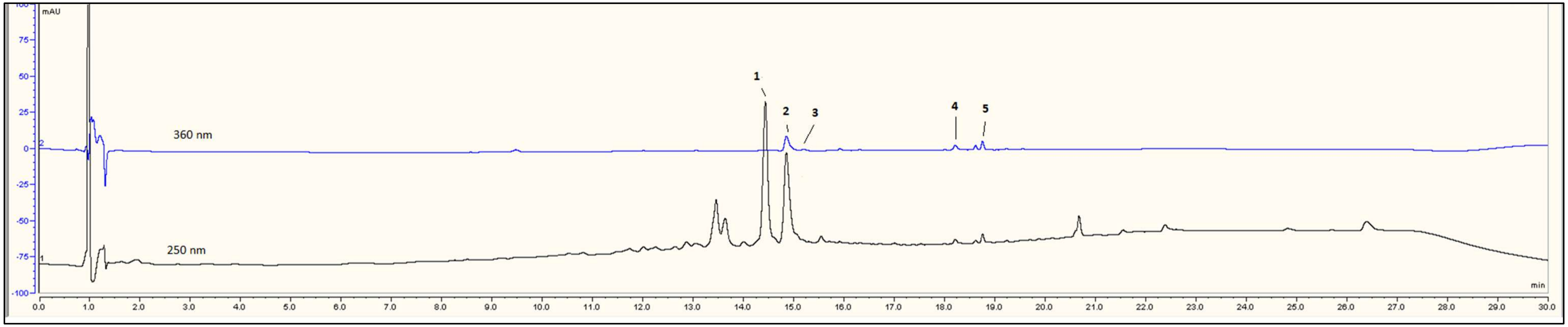

Figure 2. UV–Vis chromatograms of Rosa rugosa extract acquired at 250 and 360 nm; Peak numbers correspond to table 2 in Supplementary material.

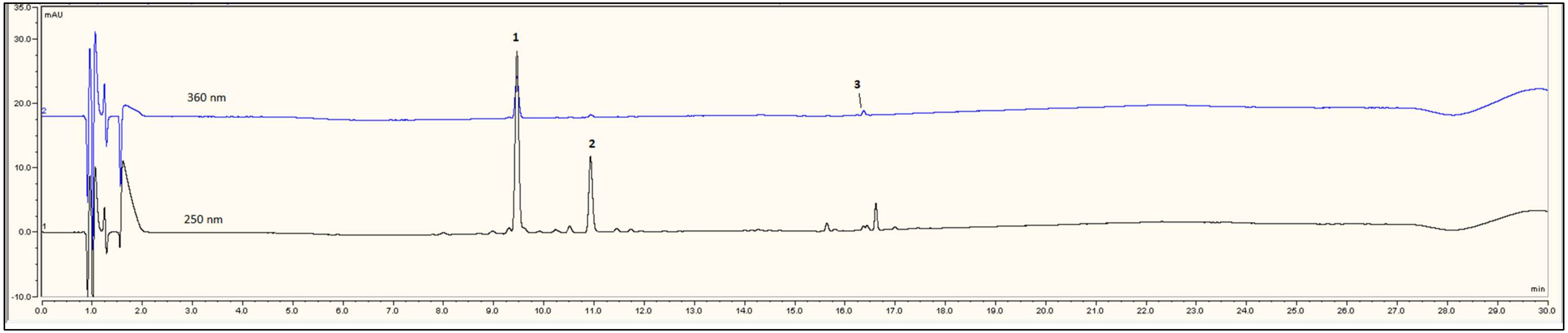

Figure 3. UV–Vis chromatograms of Champion' apples (*Malus domestica* Borkh.) acquired at 250 and 360 nm; Peak numbers correspond to table 3 in Supplementary material.
